# Cfp1 Controls Cardiomyocyte Maturation by Modifying Histone H3K4me3 of Structural, Metabolic, and Contractile Related Genes

**DOI:** 10.1002/advs.202305992

**Published:** 2024-01-09

**Authors:** Changzhu Li, Yang Zhang, Jingling Shen, Hairong Bao, Yue Zhao, Desheng Li, Sijia Li, Yining Liu, Jiming Yang, Zhiwen Zhou, Kangyi Gao, Lexin Zhao, Yao Pei, Yanjie Lu, Zhenwei Pan, Benzhi Cai

**Affiliations:** ^1^ Department of Pharmacology (State Key Laboratory of Frigid Zone Cardiovascular Disease Key Laboratory of Cardiovascular Research, Ministry of Education) College of Pharmacy Harbin Medical University Harbin Heilongjiang 150086 P. R. China; ^2^ Institute of Life Sciences College of Life and Environmental Sciences Wenzhou University Wenzhou 325035 P. R. China; ^3^ Research Unit of Noninfectious Chronic Diseases in Frigid Zone Chinese Academy of Medical Sciences 2019 Research Unit 070 Harbin Heilongjiang 150086 P. R. China; ^4^ Key Laboratory of Cell Transplantation The First Affiliated Hospital Harbin Medical University P. R. China

**Keywords:** cardiomyocyte maturation, Cfp1, H3K4me3, hiPSC‐CMs

## Abstract

Cardiomyocyte maturation is the final stage of heart development, and abnormal cardiomyocyte maturation will lead to serious heart diseases. CXXC zinc finger protein 1 (Cfp1), a key epigenetic factor in multi‐lineage cell development, remains underexplored in its influence on cardiomyocyte maturation. This study investigates the role and mechanisms of Cfp1 in this context. Cardiomyocyte‐specific Cfp1 knockout (Cfp1‐cKO) mice died within 4 weeks of birth. Cardiomyocytes derived from Cfp1‐cKO mice showed an inhibited maturation phenotype, characterized by structural, metabolic, contractile, and cell cycle abnormalities. In contrast, cardiomyocyte‐specific Cfp1 transgenic (Cfp1‐TG) mice and human induced pluripotent stem cell‐derived cardiomyocytes (hiPSC‐CMs) overexpressing Cfp1 displayed a more mature phenotype. Mechanistically, deficiency of Cfp1 led to a reduction in trimethylation on lysine 4 of histone H3 (H3K4me3) modification, accompanied by the formation of ectopic H3K4me3. Furthermore, Cfp1 deletion decreased the level of H3K4me3 modification in adult genes and increased the level of H3K4me3 modification in fetal genes. Collectively, Cfp1 modulates the expression of genes crucial to cardiomyocyte maturation by regulating histone H3K4me3 modification, thereby intricately influencing the maturation process. This study implicates Cfp1 as an important molecule regulating cardiomyocyte maturation, with its dysfunction strongly linked to cardiac disease.

## Graphical abstract

Cardiomyocyte maturation is a core process in heart development. The study highlights the critical role of CXXC zinc finger protein 1 (Cfp1) in cardiomyocyte maturation and links its dysfunction to cardiac disease. The findings reveal that Cfp1 promotes cardiomyocyte maturation by regulating trimethylation on lysine 4 of histone H3 (H3K4me3) modification of adult genes, providing potential insights into the treatment of cardiac diseases.

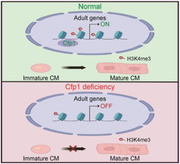

## 1 Introduction

Cardiomyocyte maturation, the transformative process from fetal to adult cardiomyocytes, involves significant changes in cell structure, metabolism, function, and gene expression.^[^ ^1^ ^]^ The whole process of cardiomyocyte maturation takes months in rodents and about six years in humans.^[^ ^2^ ^]^ Maturation makes the heart ready for powerful, efficient, and continuous pumping of blood throughout the mammalian life cycle.

Epigenetic regulation plays an important role in the process of cardiomyocyte maturation, and dysfunction of epigenetic factors is associated with cardiac developmental disorders. Polycomb repressive complex 2 (Prc2) affects the early development of mouse heart by regulating the trimethylation on lysine 27 of histone H3.^[^ ^3^ ^]^ In the postnatal heart, embryonic ectoderm development inactivation of the Prc2 core subunit can lead to fatal dilated cardiomyopathy by affecting histone deacetylase activity.^[^ ^4^ ^]^ Mutations in mixed‐lineage leukemia 2 lead to human Kabuki syndrome, characterized by defects in the atrial and ventricular septa.^[^ ^5^ ^]^ DOT1 like histone lysine methyltransferase (Dot1l) is a histone methyltransferase that can methylate lysine 79 of histone H3. Downregulation of Dot1l expression also interferes with the cardiac differentiation of human embryonic stem cells.^[^ ^6^ ^]^ Jumonji and AT‐rich interaction domain‐containing 2, a founding member of the Jumonji C family, is an important histone demethylase for cardiac trabeculation.^[^ ^7^ ^]^ Another member of the Jumonji C family, the Jumonji domain contains 3, interacts with transcription factor Isl1 and regulates cardiac differentiation of mouse embryonic stem cells.^[^ ^8^ ^]^

CXXC zinc finger protein 1 (Cfp1) is a significant epigenetic factor with a crucial role in various developmental processes. By binding to non‐methylated Cytosine‐phosphate‐Guanine (CpG) dinucleotide DNA sequences,^[^ ^3b^ ^]^ interacting with DNA methyltransferase 1, Cfp1 influences DNA methylation levels, and regulates gene transcription activation.^[^ ^3a^ ^]^ Cfp1 is also a subunit of the histone methyltransferase complex and participates in histone H3 lysine 4 methylation modification through the histone H3 lysine 4 methyltransferase complex.^[^ ^9^ ^]^ In addition, Cfp1 can interact with SWI‐independent 3histone deacetylase to affect histone acetylation modification.^[^ ^10^ ^]^ Loss of Cfp1 in mouse oocytes leads to reduced H3K4 methylation levels and overall transcriptional activity, resulting in defects in cytoskeletal lattice formation, meiosis, and maternal zygotic transition.^[^ ^11^ ^]^ Similarly, loss of Cfp1 in adult mouse hematopoietic stem cells results in severe loss of lineage defining progenitor cells and mature cells, and increased levels of apoptosis and death within 2 weeks.^[^ ^12^ ^]^ In a previous study, we demonstrated that cardiac‐specific heterozygous deletion of Cfp1 impaired sinus node function and reduced heart rate.^[^ ^13^ ^]^ Meanwhile, we observed that cardiac Cfp1 homozygous knockout mice often died at young age, which drove us to speculate that Cfp1 may participate in the regulation of cardiac maturation.

In this study, we detected the dynamic changes of Cfp1 in the developing heart, and employed cardiomyocyte‐specific Cfp1 knockout (Cfp1‐cKO) mice, cardiomyocyte‐specific Cfp1 transgenic (Cfp1‐TG) mice, and human induced pluripotent stem cell‐derived cardiomyocytes (hiPSC‐CMs) to explore the role of Cfp1 in controlling cardiomyocyte maturation and reveal its underlying mechanism. We found that Cfp1 acts as a critical regulator of the entire process of cardiomyocyte maturation and maturation maintenance by epigenetically regulating the expression of maturation‐related genes.

## 2 Results

### 2.1 Cardiomyocyte‐Specific Deletion of CXXC Zinc Finger Protein 1 (Cfp1) Leads to Immature Death in Mice

**Figure 1:**
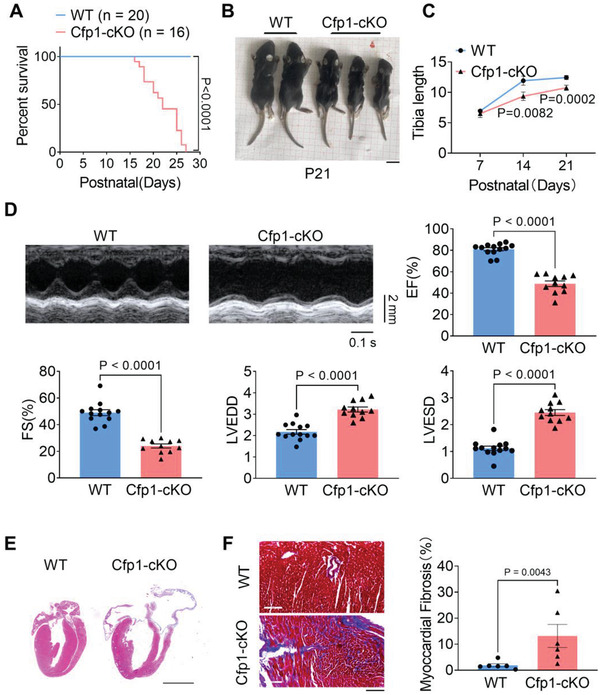
Cardiomyocyte‐specific deletion of Cfp1 leads to immature death in mice. A) Survival rate of Cfp1‐cKO mice. Simple survival analysis (Kaplan‐Meier) was used. B) Representative images of mice morphology at postnatal day 21 (P21). Scale bars: 1 cm. C) Tibia length of WT and Cfp1‐cKO mice from P7 to P21. n>5 per timepoint. D) Echocardiographic parameters of EF, FS, LVEDD and LVESD in WT and Cfp1‐cKO mice. n = 11‐13. E) Representative H&E‐stained paraffin section displays morphology of WT and Cfp1‐cKO hearts. Scale bar: 2 mm. n = 5. F) Representative Masson‐stained paraffin section display fibrosis and quantification of the relative fibrotic area of hearts from WT and Cfp1‐cKO mice. n = 6. Scale bar: 100 µm. *P* values are indicated on the graphs comparing WT versus Cfp1‐cKO. Student's t test or Mann‐Whitney U test was used. Bars represent Mean ± SEM.

The survival curve of Cfp1‐cKO mice showed that they began to die at 2 weeks after birth, and all died within four weeks (**Figure** **1**A). Additionally, Cfp1‐cKO mice exhibited stunted growth compared to wild‐type (WT) littermate controls at 3 weeks of age (Figure 1B). By observing the body weight and tibia length of the mice at different time points, we found that Cfp1‐cKO mice exhibited developmental delay starting from the 7th day after birth compared to WT mice (Figure 1C; Figure S1, Supporting Information). At 3 weeks of age, Cfp1‐cKO mice displayed impaired cardiac function, evidenced by reduced ejection fraction (EF) and fractional shortening (FS), and increased left ventricular end‐systolic diameter (LVESD) and left ventricular end‐diastolic diameter (LVEDD) (Figure 1D). Histological analyses, including hematoxylin‐eosin (H&E) and Masson's trichrome staining, revealed significant cardiac chamber enlargement, thinning of the ventricular walls, and interstitial fibrosis in 3‐week‐old Cfp1‐cKO mice (Figure 1E,F). These findings indicated that Cfp1 knockout leads to significant abnormalities in cardiac morphology and function, ultimately resulting in mouse mortality.

### 2.2 Cardiomyocyte‐Specific Deletion of Cfp1 Alters Expression Pattern of Maturation Related Genes

**Figure 2:**
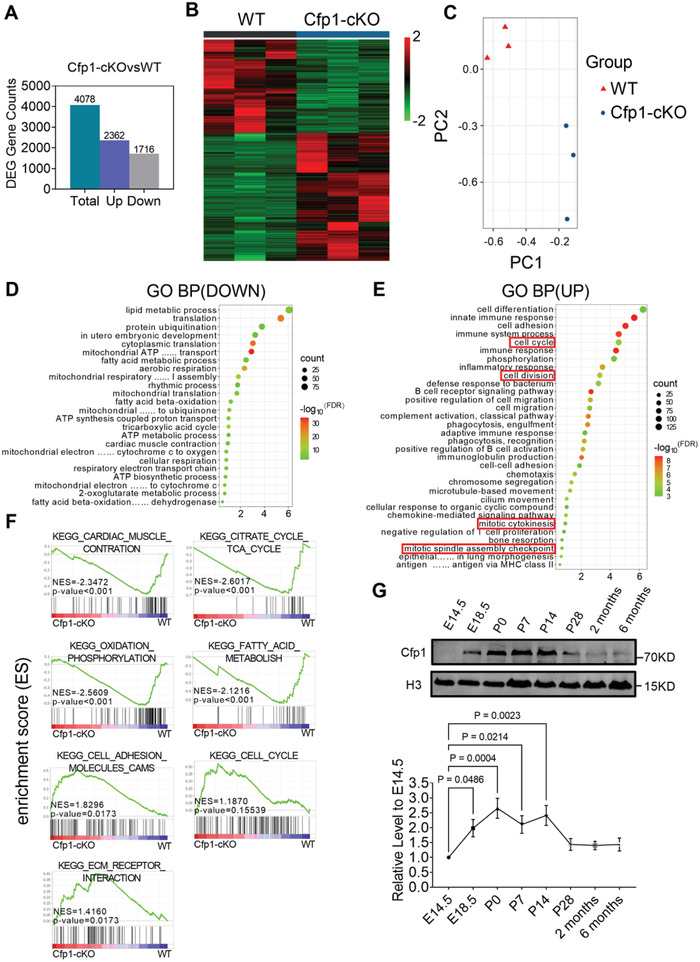
Cardiomyocyte‐specific deletion of Cfp1 alters expression pattern of maturation related genes. A) The number of DEGs (*P‐adjust*<0.05 and |log_2_^FC^|>0.5), up‐regulated DEGs and down‐regulated DEGs. B) Heatmap depicting normalized RNA‐seq expression values of DEGs. Gene regulations are reported as a color code and hierarchical clustering as a dendrogram. C) Principal component analysis performed on rlog‐normalized counts for all RNA‐seq samples. D) GO‐BP analysis of up‐regulated genes in DEGs. Bonferroni *P* < 0.05. E) GO‐BP analysis of down‐regulated genes in DEGs. Bonferroni *P* < 0.05. F) Representative enrichment plots from gene set enrichment analysis. Normalized enrichment score (NES) and *P* value are specified. G) Western blot analysis of Cfp1 protein expression at different time points. From embryonic day 14.5 (E14.5) to 6 months, n = 5 per timepoint. One‐way analysis of variance (ANOVA) followed by Dunnett multiple comparisons test versus E14.5, *P* values are indicated on the graphs. All bars represent mean ± SEM.

To explore the influence of Cfp1 on cardiac gene transcription, we performed RNA sequencing (RNA‐seq) on hearts of 2‐week‐old Cfp1‐cKO mice. Compared to WT controls, a total of 4078 differentially expressed genes (DEGs) were identified in Cfp1‐cKO mice, with 2362 upregulated and 1716 downregulated (**Figure** **2**A,B). Principal component analysis effectively distinguished between Cfp1‐cKO and WT mice (Figure 2C). Gene ontology biological process (GO‐BP) analysis of DEGs revealed that upregulated genes were enriched in biological processes related to cell division and the cell cycle, while downregulated genes were enriched in biological processes associated with energy metabolism and cardiac contraction (Figure 2D,E). Gene set enrichment analysis showed that the calcium signal pathway, cardiac muscle contraction pathway, fatty acid oxidation (FAO) pathway, oxidative phosphorylation (OXPHOS) pathway, and tricarboxylic acid cycle pathway were down‐regulated, whereas cell cycle pathway, cell adhesion molecules pathway, and ECM receptor interaction pathway was up‐regulated (Figure 2F). These data indicated that Cfp1 knockout affects the expression of maturation‐related genes in cardiomyocytes.^[^ ^14^ ^]^

Then, examining the expression of Cfp1 at different time points in the heart, we noted elevated levels during the perinatal period, a critical phase for cardiomyocyte maturation (Figure 2G).^[^ ^15^ ^]^ This temporal pattern suggested a potential influential role for Cfp1 in the process of cardiomyocyte maturation.

### 2.3 Cardiomyocyte‐Specific Deletion of Cfp1 Impairs Cardiomyocyte Maturation

**Figure 3:**
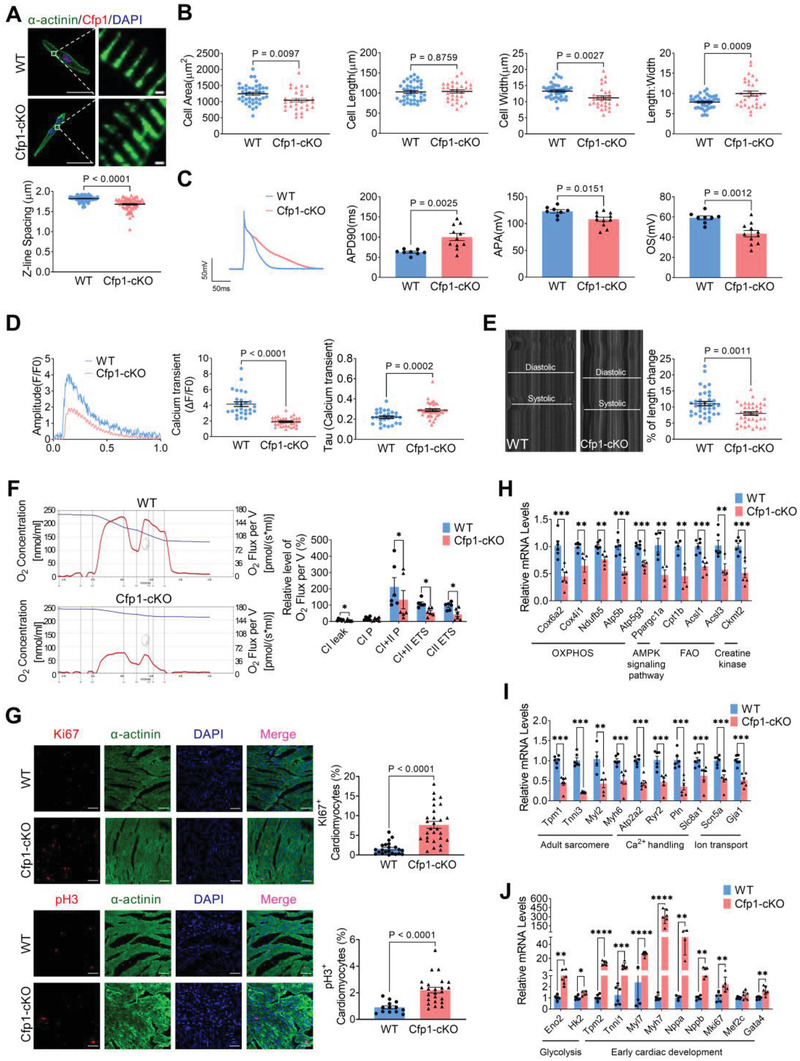
Cardiomyocyte‐specific deletion of Cfp1 impairs cardiomyocyte maturation. A) Immunofluorescence representative map of α‐actinin and demonstrating spacing of sarcomere z‐lines. n = 69‐75 cells from at least 3 mice. Scale bar: 50 µm (left); Scale bar: 1 µm (right). *P* values are indicated on the graphs comparing WT versus Cfp1‐cKO. B) Quantification of the size and morphology of isolated CMs by cell area, cell length, cell width and length/width. n = 30‐41 cells from at least 3 mice. *P* values are indicated on the graphs comparing WT versus Cfp1‐cKO. C) Representative tracing of AP by whole‐cell patch clamp recording and quantification of APD_90_, APA, and OS. n = 8‐11 cells from at least 3 mice. P values are indicated on the graphs comparing WT versus Cfp1‐cKO. D) Representative traces of calcium transient peak calcium transient (ΔF/F0) and Tau value of the recovery phase of calcium transient in isolated cardiomyocytes from WT and Cfp1‐cKO hearts. n = 27‐36 cells from at least 3 mice. *P* values are indicated on the graphs comparing WT versus Cfp1‐cKO. E) Representative diastolic and systolic recording of isolated cardiomyocytes and shortening of isolated single cardiomyocyte. n = 37‐39 cells from at least 3 mice. *P* values are indicated on the graphs comparing WT versus Cfp1‐cKO. F) The representative profile of O_2_ concentration change and relative level of O_2_ flux per volume and summarized data of mitochondrial respiration, including CI leak, CI P, CI+II P, CI+II ETS and CII ETS. n = 6. G) Immunofluorescence staining of Ki67 and pH3 and quantification of positive cells. Scale bar: 50 µm. n = 13‐28 from at least 3 mice. *P* values are indicated on the graphs comparing WT versus Cfp1‐cKO. H) Levels of mRNA transcripts encoding fatty acid aerobic metabolic genes, and I,J) sarcomere proteins, ion channels, glycolysis and early cardiac development genes in ventricles from WT and Cfp1‐cKO mice hearts as measured by quantitative Real‐Time PCR (qRT‐PCR). n = 4‐6. *P< 0.05, **P< 0.01, ***P< 0.001, ****P< 0.0001 versus WT. Student's t test or Mann‐Whitney U test was used. Bars represent the mean ± SEM. Atp5b, ATP synthase F1 subunit beta; Atp5g3, ATP synthase membrane subunit c locus 3.

To explore the influence of Cfp1 deficiency on cardiomyocyte maturation, we evaluated the morphological structure, electrical, contractile, and metabolic function of 2‐week‐old mouse cardiomyocytes, as well as their cell cycle. Immunofluorescent staining showed that Cfp1‐cKO mice had shorter sarcomere length, smaller cell area, normal cell length, shorter cell width, and a greater length‐to‐width ratio (**Figure** **3**A,B). Patch clamp recording showed that cardiomyocyte‐specific Cfp1 deletion led to prolongation of action potential duration at 90% repolarization (APD_90_), reduced overshoot (OS), and decreased action potential amplitude (APA) (Figure 3C). Cardiomyocytes from Cfp1‐cKO mice showed a smaller amplitude of peak systolic Ca^2+^ transients and a longer time course (τd) of Ca^2+^ transient decay during the contractile phase compared to WT mice (Figure 3D). Cardiomyocytes from Cfp1‐cKO mice showed a smaller percentage of length changes during contraction compared to WT mice (Figure 3E). We further examined the mitochondrial respiration related to Complex I and II. Mitochondrial respiration function was assessed through the respiratory leak state of complex I (CI Leak), complex I OXPHOS (CI P) capacity, complex I and II OXPHOS (CI+II P) capacity, complex I and II electron transport system (CI+II ETS), and Complex II electron transport system (CII ETS). Compared with WT mice, Cfp1‐cKO mice showed an overall reduction in mitochondrial function as the CI leak, CI+II P, CI+II ETS, and CII ETS parameters were markedly reduced (Figure 3F). The positive rate of Ki67 and pH3 was increased in the ventricles of Cfp1‐cKO mice, indicating increased cardiomyocyte proliferation (Figure 3G).

Lately, we found that the expression of genes involved in multiple mitochondrial energy transduction pathways, including FAO (Cpt1b, carnitine palmitoyltransferase 1B; Acsl1, acyl‐CoA synthetase long chain family member 1; Acsl3, acyl‐CoA synthetase long chain family member 3; etc), and OXPHOS (Cox6a2, cytochrome c oxidase subunit 6A2; Cox4i1, cytochrome c oxidase subunit 4I1; Ndufb5, NADH: ubiquinone oxidoreductase subunit B5; etc), were highly downregulated in the hearts of Cfp1‐cKO mice. In addition, expression of genes related to AMPK signaling pathway (Ppargc1a, PPARG coactivator 1 alpha) and Creatine kinase (Ckmt2, creatine kinase, mitochondrial 2) were significantly decreased (Figure 3H). Consistent with the reduced expression of genes involved in mitochondrial oxidative metabolism, a subset of glycolytic genes, including enolase 2 (Eno2) and hexokinase 2 (Hk2) were induced, consistent with persistent glycolytic metabolism, characteristic of the fetal heart (Figure 3J). Apart from downregulated mitochondrial and energy metabolism pathways, genes related to cardiac structure, including adult contractile protein gene subtypes, were affected. In Cfp1‐cKO mouse hearts, the expression of tropomyosin 1 (Tpm1), troponin I3 (Tnni3), myosin light chain 2 (Myl2), and myosin heavy chain 6 (Myh6) was reduced, while the levels of fetal isoforms tropomyosin 2 (Tpm2), troponin I1 (Tnni1), myosin light chain 7 (Myl7), and myosin heavy chain 7 (Myh7) were upregulated (Figure 3I,J). The expression of Ca^2+^ handing and ion channel/transporter genes, including ATPase sarcoplasmic/endoplasmic reticulum Ca^2+^ transporting 2 (Atp2a2), ryanodine receptor 2 (Ryr2), phospholamban (Pln), solute carrier family 8 member A1 (Slc8a1), sodium voltage‐gated channel alpha subunit 5 (Scn5a), and gap junction protein alpha 1 (Gja1), were also reduced (Figure 3I). Moreover, genes related to early heart development, such as natriuretic peptide A (Nppa), natriuretic peptide B (Nppb), marker of proliferation Ki‐67 (Mki67), myocyte enhancer factor 2C (Mef2c), and GATA binding protein 4 (Gata4), showed upregulated levels (Figure 3J).

### 2.4 Cardiomyocyte‐Specific Overexpression of Cfp1 Promotes Cardiomyocyte Maturation

**Figure 4:**
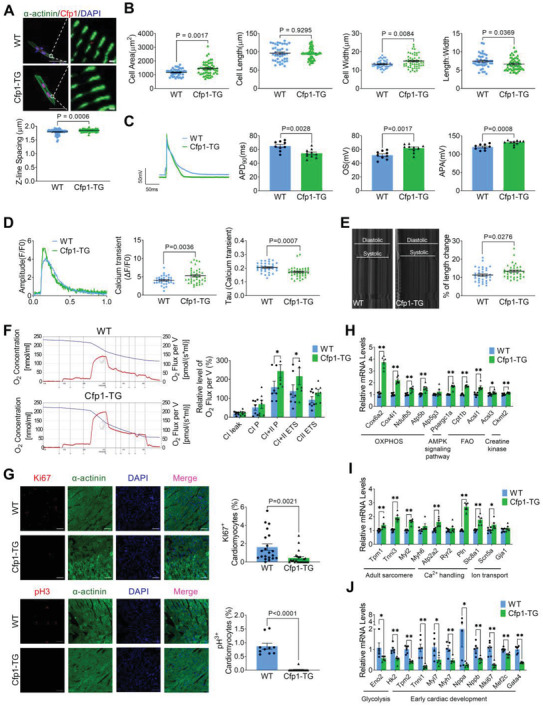
Cardiomyocyte‐specific overexpression of Cfp1 promotes cardiomyocyte maturation. A) Immunofluorescence representative map of α‐actinin and demonstrating spacing of sarcomere z‐lines. n = 60‐65 cells from at least 3 mice. Scale bar: 50 µm (left); Scale bar: 1 µm (right). *P* values are indicated on the graphs comparing WT versus Cfp1‐TG. B) Quantification of the size and morphology of isolated CMs by cell area, cell length, cell width and length/width. n = 41‐55 cells from at least 3 mice. *P* values are indicated on the graphs comparing WT versus Cfp1‐TG. C) Representative tracing of AP by whole‐cell patch clamp recording and quantification of APD90, APA, OS. n = 9‐10 cells from at least 3 mice. *P* values are indicated on the graphs comparing WT versus Cfp1‐TG. D) Representative traces of calcium transient peak calcium transient (ΔF/F0) and Tau value of the recovery phase of calcium transient in isolated cardiomyocytes from WT and Cfp1‐TG hearts. n = 32‐37 cells from at least 3 mice. *P* values are indicated on the graphs comparing WT versus Cfp1‐TG. E) Representative diastolic and systolic recording of isolated cardiomyocytes and shortening of isolated single cardiomyocyte. n = 33‐35 cells from at least 3 mice. *P* values are indicated on the graphs comparing WT versus Cfp1‐TG. F) The representative profile of O_2_ concentration change and relative level of O_2_ flux per volume and summarized data of mitochondrial respiration, including CI leak, CI P, CI+CII P, CI+CII ETS and CII ETS. n = 7. G) Immunofluorescence staining of Ki67 and pH3 and quantification of positive cells. Scale bar: 50 µm. n = 10‐24 from at least 3 mice. *P* values are indicated on the graphs comparing WT versus Cfp1‐TG. H) Levels of mRNA transcripts encoding fatty acid aerobic metabolic genes, and I,J) sarcomere proteins, ion channels, glycolysis and early cardiac development genes in ventricles from WT and Cfp1‐TG mice hearts as measured by qRT‐PCR. n = 4‐6. *P< 0.05, **P< 0.01 versus WT. Student's t test or Mann‐Whitney U test was used. Bars represent the mean ± SEM.

To further validate the influence of Cfp1 on cardiomyocyte maturation, we generated Cfp1‐TG mice (Figure S2, Supporting Information). Cardiomyocyte morphology, electrical, contractile and metabolic functions, as well as cell cycle were also assessed at 2 weeks of age. Cardiomyocytes of Cfp1‐TG mice had longer sarcomere length, larger cell area, normal cell length, longer cell width, and a smaller length‐to‐width ratio (**Figure** **4**A,B). Transgenic overexpression of Cfp1 in mice shortened APD_90_ and increased OS and APA of cardiomyocytes (Figure 4C). Meanwhile, compared to WT mice, the amplitude of the peak systolic Ca^2+^ transient was larger, and the time course for the decay phase of the Ca^2+^ transient (τd) was shorter in Cfp1‐TG mice (Figure 4D). Additionally, cardiomyocytes from Cfp1‐TG mice showed a greater percentage of length changes during contraction compared to WT mice (Figure 4E). Compared with WT mice, Cfp1‐TG mice had an overall increase in mitochondrial function, and CI+II P and CI+II ETS parameters were significantly increased (Figure 4F). The positive rate of Ki67 and pH3 in the ventricles of Cfp1‐TG mice was significantly lower than that of WT mice, indicating that Cfp1‐TG mice have reduced cardiomyocyte proliferation (Figure 4G). In Cfp1‐TG mice, the expression of genes related to FAO, OXPHOS, contraction, ion channels and calcium homeostasis increased (Figure 4H,I). Meanwhile, genes related to glycolysis and early fetal cardiac development showed reduced expression (Figure 4J).

### 2.5 Influence of Cfp1 on Late Fetal Maturation of Mouse Heart

**Figure 5:**
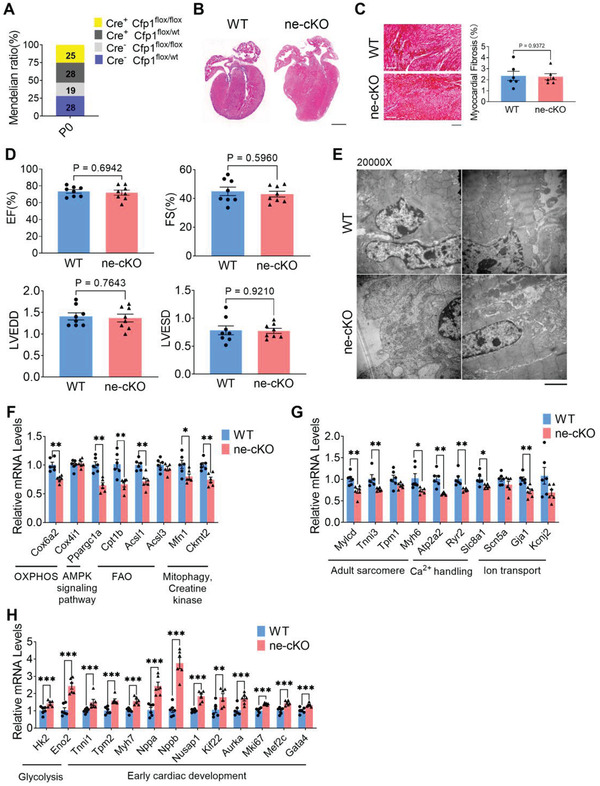
Influence of Cfp1 on late fetal maturation of mouse heart. A) Progeny number of the four genotypes (Cfp1^flox/wt^, Myh6‐Cre: Cfp1^flox/wt^, Cfp1^flox/flox^ and Myh6‐Cre: Cfp1^flox/flox^) from paternal (Myh6‐Cre: Cfp1^flox/wt^) and maternal (Cfp1^flox/flox^) breeding at postnatal day 0 (P0), respectively. B) Representative H&E‐stained paraffin section displays morphology of WT and ne‐cKO hearts. Scale bar: 500 µm. n = 5. C) Representative Masson‐stained paraffin section display fibrosis and quantification of the relative fibrotic area of hearts from WT and ne‐cKO mice. Scale bar: 100 µm. n = 6. P values are indicated on the graphs comparing WT versus ne‐cKO. D) Quantification of EF, FS, LVEDD and LVESD. n = 8. P values are indicated on the graphs comparing WT versus ne‐cKO. E) Representative electron microscope images of left ventricular wall of WT and ne‐cKO hearts (×20 000 magnification). Scale bars: 2 µm. F) Levels of mRNA transcripts encoding fatty acid aerobic metabolic genes, and G,H) sarcomere proteins, ion channels, glycolysis and early cardiac development genes in ventricles from WT and ne‐cKO mice hearts as measured by qRT‐PCR. n = 4‐6. *P < 0.05, **P < 0.01, ***P < 0.001, ****P < 0.0001 versus WT. Student's t test or Mann‐Whitney U test was used. Bars represent the mean ± SEM. Mfn1, mitofusin 1; Mylcd, malonyl‐CoA decarboxylase; Kcnj2, potassium inwardly‐rectifying channel, subfamily J, member 2; Nusap1, nucleolar and spindle associated protein 1; Kif22, kinesin family member 22; Aurka, aurora kinase A.

As the expression level of Cfp1 gradually increased from E14.5 to P0 days (Figure 2G), we next explored the influence of Cfp1 on cardiomyocyte maturation of neonatal mice. We calculated the ratio of neonatal mice of four different genotypes of Myh6‐Cre: Cfp1^flox/wt^ and Cfp1^flox/flox^ mice in the paternal and maternal generations, and found that the ratio was 1:1:1:1, fully in line with Mendelian inheritance laws (**Figure** **5**A), which implies that Cfp1 deficiency did not cause prenatal lethality.

Neonatal Cfp1‐cKO (ne‐cKO) mice showed no differences in cardiac morphology, fibrosis levels, and cardiac function compared to WT mice (Figure 5B–D). However, electron microscopy images showed a reduced number and volume of mitochondria in the left ventricle wall of ne‐cKO hearts compared with WT hearts (Figure 5E). Similarly, the expression of genes related to FAO, OXPHOS, contraction, ion channels and calcium homeostasis were decreased in ne‐cKO mice (Figure 5F,G). Meanwhile, ne‐cKO mice displayed the persistent activation of markers related to early fetal development, including fetal cardiac/skeletal muscle isoforms, glycolysis, and the cell cycle (Figure 5H). These data indicated that gene expression changes induced by Cfp1 deficiency in neonatal mice occur earlier than morphological changes.

### 2.6 Cfp1 Affects Cardiomyocyte Maturation by Modifying Trimethylation on Lysine 4 of Histone H3

**Figure 6:**
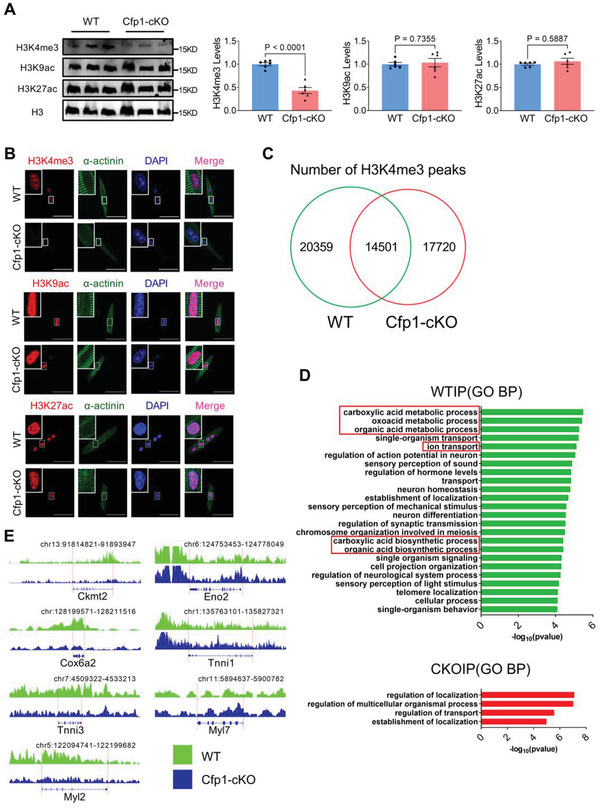
Cfp1 affects cardiomyocyte maturation by modifying histone H3K4me3. A) Protein levels of H3K4me3, H3K9ac, H3K27ac in ventricles from WT and Cfp1‐cKO mice by western blot. H3 was used as loading control. n = 6. B) Immunofluorescent staining performed to analyze the levels of H3K4me3, H3K9ac and H3K27ac in isolated ventricle cardiomyocytes from WT and Cfp1‐cKO. Scale bar: 50 µm. n = 20‐38 cells from at least 3 mice. C) The number of shared peaks and specific peaks obtained across different samples using a Venn diagram. D) GO‐BP analysis of genes with H3K4me3 peaks observed in the hearts of WT and Cfp1‐cKO mice. E) H3K4me3 modification of maturation related genes (Ckmt2, Cox6a2, Tnni3, Myl2, Eno2, Tnni1 and Myl7) by IGV. n = 1 from at least 3 mice heart. *P* values are indicated on the graphs comparing WT versus Cfp1‐cKO. Student's t test or Mann‐Whitney U test was used. Bars represent the mean ± SEM.

Cfp1 can modulate gene expression by affecting the modification of histone trimethylation on lysine 4 of histone H3 (H3K4me3), acetylation on lysine 9 of histone H3 (H3K9ac) and acetylation on lysine 27 of histone H3 (H3K27ac).^[^ ^13^, ^16^ ^]^ We found that histone H3K4me3 modification was markedly reduced in the hearts of 2‐week‐old Cfp1‐cKO mice, while upregulated in Cfp1‐TG mice (**Figure** **6**A,B; Figure S3, Supporting Information). The level of H3K9ac and H3K27ac modifications did not change in the hearts of 2‐week‐old Cfp1‐cKO or Cfp1‐TG mice (Figure 6A,B; Figure S3, Supporting Information). Sha Q et al. showed that changes in H3K4me3 modification levels were accompanied by reverse changes in monomethylation on lysine 4 of histone H3 (H3K4me1) and dimethylation on lysine 4 of histone H3 (H3K4me2) modification levels.^[^ ^17^ ^]^ In the present study, we examined the levels of H3K4me1 and H3K4me2 modifications in cardiomyocytes from Cfp1‐cKO and Cfp1‐TG mice. The levels of H3K4me1 and H3K4me2 were up‐regulated in Cfp1‐cKO mice, and down‐regulated in Cfp1‐TG mice (Figure S4, Supporting Information). We conducted Chromatin immunoprecipitation and sequencing (ChIP‐seq) on 2‐week‐old Cfp1‐cKO and WT mice. Cfp1 deletion led to the disappearance of 20359 pre‐existing H3K4me3 peaks and generated 17720 new H3K4me3 peaks (Figure 6C). GO analysis of the specific H3K4me3 peak modified genes in the two groups showed that the genes involved in biological processes such as organic acid metabolism and ion transport were absent in the hearts of Cfp1‐cKO mice (Figure 6D). In addition, genes related to mitochondrial aerobic metabolism pathways, such as Cox6a2 and Ckmt2, showed reduced H3K4me3 modification intensity in the Cfp1‐cKO group. In contrast, glycolysis‐related genes including Eno2 showed enhanced H3K4me3 modification in Cfp1‐cKO group (Figure 6E). At the same time, compared with the WT group, the Cfp1‐cKO group showed changes in H3K4me3 modification of cardiomyocytes structure related genes, which showed that the H3K4me3 modification intensity of adult isoforms Tnni3 and Myl2 genes was decreased, and the H3K4me3 modification intensity of fetal isoforms Tnni1 and Myl7 genes was increased (Figure 6E).

### 2.7 Cardiomyocyte‐Specific Deletion of Cfp1 in Adult Mice Impairs Mitochondrial Function and Leads to Heart Failure

**Figure 7:**
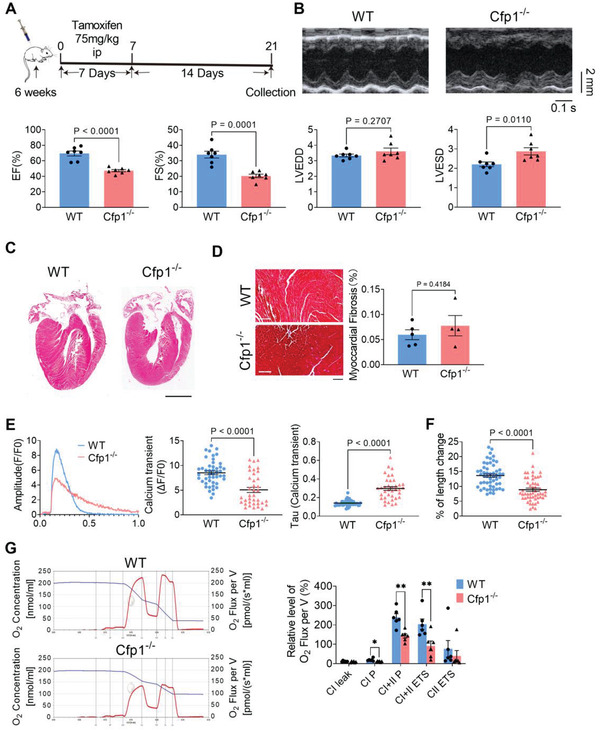
Cardiomyocyte‐specific deletion of Cfp1 in adult mice impairs mitochondrial function and leads to heart failure. A) Schematic showing the experimental timeline. B) Echocardiographic data of EF, FS, LVEDD and LVESD of WT and Cfp1^−/‐^ mice. n = 7. *P* values are indicated on the graphs comparing WT versus Cfp1^−/−^. C) Representative H&E‐stained paraffin section displays morphology of WT and Cfp1^−/−^ hearts. Scale bar: 500 µm. n = 5. D) Representative Masson‐stained paraffin section display fibrosis and quantification of relative fibrotic area of WT and Cfp1^−/−^ mice hearts. Scale bar: 100 µm. n = 4‐5. *P* values are indicated on the graphs comparing WT versus Cfp1^−/−^. E) Change in the peak calcium transient (ΔF/F0) and Tau value of the recovery phase of calcium transient in isolated ventricular myocytes. n = 37‐46 cells from at least 3 mice. *P* values are indicated on the graphs comparing WT versus Cfp1^−/−^. F) Shortening of isolated single ventricular myocytes. n = 53 cells from at least 3 mice. *P* values are indicated on the graphs comparing WT versus Cfp1^−/−^. G) The representative profile of O_2_ concentration change and relative level of O_2_ flux per volume and summarized data of mitochondrial respiration, including CI leak, CI P, CI+CII P, CI+CII ETS and CII ETS. n = 6. *P < 0.05, **P < 0.01 versus WT. Student's t test or Mann‐Whitney U test was used. Bars represent the mean ± SEM.

We further explored the impact of Cfp1 deficiency on adult heart. Tamoxifen‐induced cardiomyocyte‐specific Cfp1 knockout (Cfp1^−/−^) mice were injected intraperitoneally with tamoxifen (75 mg kg^−1^) for 7 consecutive days at 6 weeks of age, and functional testing was performed 14 days later (**Figure** **7**A). Cfp1 protein and mRNA levels were significantly reduced in the hearts of Cfp1^−/−^ mice, indicating successful induction of Cfp1 deletion in adult mice (Figure S5A,B, Supporting Information). Echocardiography results showed a significant decrease in EF and FS, an increase in LVESD, but no change in LVEDD (Figure 7B). H&E and Masson's trichrome staining showed that there were no obvious differences in cardiac morphology and interstitial fibrosis between Cfp1^−/−^ and WT mice (Figure 7C,D). The amplitude of peak systolic Ca^2+^ transient was smaller in cardiomyocytes from Cfp1^−/−^ than WT mice, and the time course for the decay phase of Ca^2+^ transient (τd) was longer in Cfp1^−/−^ than WT mice (Figure 7E). Cardiomyocytes from Cfp1^−/−^ mice showed a smaller percentage of length changes during contraction compared to WT mice (Figure 7F). The hearts of Cfp1^−/−^ mice also showed an overall reduction in mitochondrial function and indicated by reduction in CI P, CI+II P and CI+II ETS parameters (Figure 7G). These data implied that deficiency of Cfp1 in adult mice also impairs the contractile and mitochondrial function of cardiomyocytes.

### 2.8 Overexpression of Cfp1 Promotes Human Induced Pluripotent Stem Cell‐Derived Cardiomyocytes Maturation

**Figure 8:**
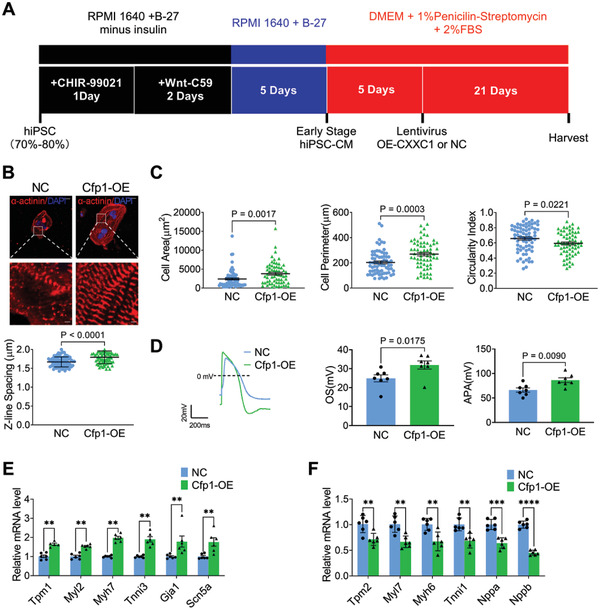
Overexpression of Cfp1 promotes hiPSC‐CMs maturation. A) Schematic graph showing the experimental timeline. B) Immunofluorescent staining of α‐actinin and spacing of sarcomere z‐lines. n = 71‐82 cells. Scale bar: 20 µm (top); Scale bar: 2 µm (down). *P* values are indicated on the graphs comparing NC versus Cfp1‐OE. C) Quantification of the size and morphology of hiPSC‐CMs. n = 63‐70 cells. *P* values are indicated on the graphs comparing NC versus Cfp1‐OE. D) Representative tracing of action potential by whole‐cell patch‐clamp technique and quantification of recorded APA and OS. n = 7 cells. *P* values are indicated on the graphs comparing NC versus Cfp1‐OE. E,F) Levels of mRNA transcripts encoding adult genes, and fetal genes as measured by qRT‐PCR. n = 5‐6. **P < 0.01, ***P < 0.001, ****P < 0.0001 versus NC. Student's t test or Mann‐Whitney U test was used. Bars represent the mean ± SEM. hiPSC, human induced pluripotent stem cell.

To further validate the influence of Cfp1 on cardiomyocyte maturation, we examined its effect on the maturation of hiPSC‐CMs. We infected hiPSC‐CMs with lentivirus carrying Cfp1 overexpression plasmid and detected the maturation of hiPSC‐CMs after 3 weeks (**Figure** **8**A). Cfp1 levels in the Cfp1 overexpression (Cfp1‐OE) group were significantly higher than in the negative control (NC) group (Figure S5C, Supporting Information). Immunofluorescent staining showed that overexpression of Cfp1 increased sarcomere length, cell area and perimeter of hiPSC‐CMs, while decreasing cell circularity index (Figure 8B,C). Cfp1 overexpression increased OS and APA of hiPSC‐CMs (Figure 8D). Overexpression of Cfp1 in hiPSC‐CMs resulted in increased expression of human adult myosin isoforms (Tpm1, Myl2, Myh7, and Tnni3) and ion transport (Gja1 and Scn5a) genes. Human fetal myosin isoforms (Tpm2, Myl7, Myh6, and Tnni1) and natriuretic peptide (Nppa, and Nppb) were decreased (Figure 8E,F). These data indicated that overexpression of Cfp1 promotes the maturation of hiPSC‐CMs.

## 3 Discussion

Cfp1 is a crucial epigenetic factor that plays an essential role in achieving developmental competence.^[^ ^18^ ^]^ Global knockout of Cfp1 in mice leads to embryonic lethality,^[^ ^19^ ^]^ and the deletion of Cfp1 in various cell types results in developmental and maturation impairment.^[^ ^16^, ^20^ ^]^ In the present study, it was observed that Cfp1 expression levels in the heart were elevated during the perinatal period, which corresponds to a critical stage in cardiomyocyte maturation. Cfp1‐cKO mice started to die at 2 weeks after birth and all died within 4 weeks. Cfp1 deficiency results in impaired cardiomyocyte maturation, evidenced by alterations in cardiomyocyte morphology and structure, cardiac electrical activity, contractile and metabolic functions, and cardiomyocyte proliferation. These findings suggested that Cfp1 is a key regulator of cardiomyocyte maturation.

Several epigenetic modifications are associated with transcriptionally active euchromatin or repressed heterochromatin. Histone methylation and acetylation modifications are associated with transcriptionally active euchromatin.^[^ ^21^ ^]^ H3K4me3, H3K9ac, and H3K27ac are critical epigenetic modifications involved in the regulation of various cardiac diseases.^[^ ^22^ ^]^ Zhao et al. reported that knockout of Cfp1 led to loss of H3K4me3, H3K9ac and H3K27ac modifications in the promoter of colony stimulating factor 2 receptor subunit alpha gene and reduced its expression.^[^ ^16^ ^]^ In a previous study, our group demonstrated that cardiomyocyte specific Cfp1 deletion caused a decrease in hyperpolarization activated cyclic nucleotide gated potassium channel 4 (Hcn4) transcription and reduced cardiac rhythm due to loss of H3K4me3, H3K9ac, and H3K27ac modifications in the Hcn4 gene promoter.^[^ ^13^ ^]^ In the present study, we examined changes in H3K4me3, H3K9ac, and H3K27ac modification in Cfp1‐cKO mice and found that only H3K4me3 was downregulated. Furthermore, cardiomyocyte specific overexpression of Cfp1 also only led to upregulation of H3K4me3 modification. Previous studies have shown that Cfp1 can affect the level of H3K4me3 modification in cells. Tate et al. showed that murine embryonic stem cells lacking Cfp1 results in a global H3K4me3 upregulation.^[^ ^23^ ^]^ In contrast, Clouaire et al. showed that Cfp1 is required for H3K4me3 and deficiency of Cfp1 in embryonic stem cells leads to marked loss of H3K4me3 at expressed CpG island associated genes.^[^ ^24^ ^]^ Sha Q et al. found that Cfp1 and H3K4me3 change in the same direction during meiotic maturation.^[^ ^17^ ^]^ In addition, in the present study, we found that the level of H3K4me3 modification is significantly altered in the hearts of Cfp1 knockout and overexpression mice, but the levels of H3K9ac and H3K27ac modification were not altered. Therefore, we thought that H3K4me3 might be the key epigenetic modification affecting cardiomyocyte maturation and analyzed H3K4me3 modification sites, but not H3K9ac and H3K27ac modification sites. The possibility that the overall level of H3K9ac and H3K27ac modification is unchanged, while specific local modification sites changed, cannot be excluded.

Epigenetic regulation plays a crucial role in embryonic development and cell type‐specific control of gene expression. However, the specific impact of histone methylation changes on cardiac differentiation and maturation has remained less explored. Existing research has demonstrated that increasing H3K4me3 modifications on the promoters of genes in human embryonic stem cells and their derived ventricular cardiomyocytes induces hypertrophic growth of cardiomyocytes and enhances the expression of cardiac genes such as Myh6, Myh7, and Myl2.^[^ ^25^ ^]^ Additionally, increased H3K4me3 modification in genes involved in mitochondrial biogenesis regulatory pathways leads to an increased rate of mature cardiomyocytes.^[^ ^26^ ^]^ In this study, we found that cardiomyocyte‐specific Cfp1 deletion results in decreased H3K4me3 modifications on genes upregulated during cardiomyocyte maturation, including Ckmt2, Cox6a2, Tnni3, and Myl2, and increased H3K4me3 modifications on genes downregulated during cardiomyocyte maturation, including Eno2, Tnni1 and Myl7. These findings suggested that Cfp1 affects the expression of genes related to cardiomyocyte maturation by regulating H3K4me3 modification, thus participating in the process of cardiomyocyte maturation.

During the transition from the fetal stage to adulthood, the heart undergoes dramatic metabolic and structural maturation. Many adult cardiac processes revert to a fetal format during the development of heart failure.^[^ ^14b^ ^]^ To further clarify the role of Cfp1 on cardiomyocyte maturation, we examined the cardiac morphology and function as well as the expression status of maturation related genes in ne‐cKO mice and found no obvious differences on cardiac structure and function, but marked abnormalities in the expression pattern of maturation related genes. These findings established that Cfp1 plays a role in the pathogenesis of heart failure by affecting cardiomyocyte maturation rather than reversing the fetal form into an adult cardiac process.

Cfp1 plays an important role in cardiomyocyte maturation and development, and the expression of Cfp1 is downregulated after perinatal period. To further elucidate the function of Cfp1, we showed that cardiomyocyte specific Cfp1 deletion in adult mice attenuates cardiac function without altering morphological structure, with significant attenuation of energy metabolism and calcium handling capacity. These findings indicated that although the expression level of Cfp1 appears significantly downregulated in adult cardiomyocytes compared with that in perinatal period, Cfp1 is still essential for the maintenance of basic functions of cardiomyocytes, indicating that Cfp1 is a key factor in cardiomyocytes and plays important roles at different stages.

Increasing H3K4me3 modifications on human embryonic stem cell‐derived ventricular cardiomyocytes gene promoters induces cardiomyocyte hypertrophic growth.^[^ ^25^ ^]^ In the present study, we constructed mice with cardiomyocyte specific overexpression of Cfp1 and found that Cfp1 overexpression promoted cardiomyocyte maturation, exhibited cell morphology and structure, and altered cardiac electrical activity, contractile and metabolic functions, and cell proliferation status. Recently, hiPSC‐CMs technology has shown considerable clinical value, but so far fully mature cardiomyocytes still cannot be obtained in vitro, and this maturation obstacle of cardiomyocytes severely limits its clinical application.^[^ ^27^ ^]^ We found that overexpression of Cfp1 promoted hiPSC‐CMs maturation. The findings further confirmed the critical role of Cfp1 in cardiomyocyte maturation.

In conclusion, the study demonstrates that Cfp1 plays an important role in the process of cardiomyocyte maturation. This function is mainly mediated by the regulation of H3K4me3 modification, which affects the expression of maturation related genes. The study provides a new direction for inducing cardiomyocyte maturation.

## 4 Experimental Section

### Animals

Myh6‐Cre transgenic C57BL/6 mice (SPF) and Cfp1^flox/flox^ transgenic C57BL/6 mice (SPF) were presented by Professor Shen Jingling, Department of Embryology, Basic Medical College of Harbin Medical University. Myh6‐MerCreMer transgenic C57BL/6 mice (SPF), as well as Myh6 promoter of Cfp1‐TG mice (SPF) were constructed by Cyagen Biosciences Co., LTD. Myh6‐Cre transgenic mice was crossed with Cfp1^flox/flox^ transgenic mice to generate Myh6‐Cre: Cfp1^flox/wt^ mice, and then crossed Myh6‐Cre: Cfp1^flox/wt^ mice with Cfp1^flox/flox^ mice again. Eventually, genotype was got for Myh6‐Cre: Cfp1^flox/flox^ Cfp1‐cKO in mice. Myh6‐MerCreMer transgenic mice was crossed with Cfp1^flox/flox^ transgenic mice to generate Myh6‐MerCreMer: Cfp1^flox/wt^ mice. Myh6‐MerCreMer: Cfp1^flox/wt^ mice were then crossed again with Cfp1^flox/flox^ mice to get Myh6‐MerCreMer: Cfp1^flox/flox^ Cfp1^−/−^ mice. The primers were synthesized by Invitrogen and listed in Table S1.

All mice were maintained in a temperature‐controlled environment with a temperature of 23 ± 3°C, humidity of 30 to 70%, and a 12‐h light/dark cycle. All animal experiments were approved by the Ethics Committee of the School of Pharmacy, Harbin Medical University (IRB3018622) and conformed to the Guide for the Care and Use of Laboratory Animals issued by National Institutes of Health (NIH Publication 85‐23, revised 1996). Experimentalists were blinded to treatment/genotype grouping information during experiments and quantification. No mice were excluded from the study unless they died.

### Construction of Lentivirus (AAV9) Carrying Cfp1

The AAV9 lentivirus carrying human Cfp1 was constructed by Genechem Co., Ltd. (Shanghai, China). The plasmid vector was GV492 and the original sequence was Ubi‐MCS‐3FLAG‐CBh‐gcGFP‐IRES‐puromycin. The overexpression gene was Cxxc1 (NM_001101654) and the negative control number was CON335.

### Echocardiography

The mice were anesthetized with 1% sodium pentobarbital, and the body temperature of mice was maintained at 36 – 37°C. The cardiac function of mice was detected by two‐dimensional M‐mode using Vevo 2000 high‐resolution small animal ultrasound imaging system. EF, FS, LVESD, and LVEDD were calculated.

### Isolation of Mouse Cardiomyocytes

Mouse cardiomyocytes were isolated as described previously.^[^ ^28^ ^]^ The mice were anesthetized with 1% sodium pentobarbital and 0.1 mL heparin (50 mg mL^−1^, ip). The heart was rapidly excised and the aorta was cannulated on a constant flow Langendorff device. The digested hearts were perfused with Tyrode solution containing 1 mg mL^−1^ collagenase type II powder, protease (0.02 mg mL^−1^), and bovine serum albumin (1 mg mL^−1^). The Tyrode's solution contained (in mM): 123 NaCl, 5.4 KCl, 10 HEPES, 0.33 NaH_2_PO_4_, 1.0 MgCl_2_, and 10 glucoses (pH adjusted to 7.4 with NaOH). After the tissue was softened, the left ventricle was gently separated into small pieces and stirred to separate the cardiomyocytes, which were then equilibrated in Tyrode's solution containing 200 µM CaCl_2_ and 1% bovine serum albumin. Single rod cells with clear transverse stripes were used for electrophysiological recordings. All solutions were gassed with 95% oxygen and 5% carbon dioxide and heated to 37±0.5 °C.

### Immunofluorescent Staining

Isolated mouse ventricular myocytes were fixed for 10 min with 4% paraformaldehyde in PBS, and then washed in PBS for 10 min (2 times). The cells were subsequently dropped onto glass slides and cells were attached to glass slides and permeabilized with 0.5% Triton X‐100 for 5 min. Frozen sections of heart tissue and cultured cells were fixed (10 min) in 4% PFA. Heart sections and cultured cells were then permeabilized with 0.5% Triton X‐100 for 5 min.

Samples were blocked with 5% bovine serum albumin (BSA) in PBS containing 0.1% Triton X‐100 for 1 h at room temperature. Then samples were incubated for 1 h at room temperature or overnight at 4 °C with the following antibodies diluted in 3% BSA blocking solution. Anti‐α‐actinin (1:500, A7811, Sigma, America) was used to identify CMs. Anti‐Ki67 (1:2000, ab15580, Abcam, America) and anti‐phosphorylated‐histone3 (pH3) (1:500, 06–570, Sigma‐Aldrich, America) antibodies were used to analyse cell‐cycle re‐entry and karyokinesis, respectively. Other antibodies used in the study: anti‐H3K4me3 (1:200, 9751S, Cell Signaling Technology, America), anti‐H3K9ac (1:200, 9649S, Cell Signaling Technology, America) and anti‐H3K27ac antibody (1:200, 8173S, Cell Signaling Technology, America). After three washes with PBS, 10 min each time, samples were stained for 1 h at room temperature or overnight at 4 °C with fluorescent secondary antibodies (Abcam) followed by 10 min of DAPI (4′,6‐diamidino‐2‐phenylindole dihydrochloride) staining for nuclei visualization. Slides were mounted with Immu‐mount (9990412, Thermo Scientific) and viewed under a fluorescence microscope (Nikon Intensilight or Nikon eclipse 90i, Nikon) or spinning‐disc confocal microscope (Carl Zeiss) as specified in the figure legends.

### Whole‐Cell Patch Clamp Recording

Whole‐cell patch clamp equipment (Axopatch 700B amplifier) was used to record action potential. Borosilicate glass electrodes with pipette resistance of 2≈4 MΩ were prepared by using a Brown‐Flamming puller (model P‐97, Sutter Instrument Co., Novato, CA., UAS) and a microforge (F‐83, Narishige, Japan). The action potentials were recorded with pipette solution (in mmol/L: K‐glutamate 130; MgCl_2_•6H_2_O 1; NaCl 5; KCl 15; Mg‐ATP 5; CaCl_2_ 1; EGTA 5; HEPES 10; pH adjusted to 7.20 with KOH) and the external solution was the normal Tyrode's solution at 37 °C.

### Measurements of Cardiac Contractility

Freshly isolated ventricular myocytes were placed in normal Tyrode's solution containing (in mM): 137 NaCl, 5.4 KCl, 0.16 NaH_2_PO_4_, 10 glucose, 1.8 CaCl_2_, 0.5 MgCl_2_, 5.0 HEPES, and 3.0 NaHCO_3_ (pH adjusted to 7.4 with NaOH) and paced to steady state using a 1 Hz field stimulation. After 20 s of stimulation, the cells showed steady contractions, and video images were acquired using a Flash4.0 LT camera by line scanning (4 ms line^−1^, C11440‐42U, Hamamatsu, Japan). Image J was used to measure the cell length under contraction (systolic length) and relaxation (diastolic length). Cardiomyocyte sarcomere shortening, which reflects the contractility of cardiac muscles, was calculated as (diastolic length–systolic length)/diastolic length × 100%. Measurements were taken from more than 20 myocytes from three or more animals from each group.

### Measurements of Intracellular Calcium Transients

To measure cellular calcium transients, both freshly isolated cardiomyocytes and primarily cultured cardiomyocytes were incubated with 5 µM Fluo‐3 (Invitrogen, Grand Island, NY, USA) and 0.01% Pluronic F127 (BASF, Florham Park, NJ, USA) in Tyrode's solution for 35 min.^[^ ^29^ ^]^ To measure intracellular Ca^2+^ transients, cardiomyocytes were electrically paced at 1 Hz. Images were obtained with an Olympus camera. The amplitude of the intracellular Ca^2+^ transient was calculated as the difference between peak and diastolic calcium levels according to the equation (F‐F0)/F0 after subtraction of background fluorescence. The calcium transient decay phase time constant (τd) was determined using exponential curve fitting. The experiments were performed at room temperature. Measurements were taken from more than 20 myocytes from three or more animals from each group.

### Mitochondrial Respiratory Function Detection

Mitochondrial respiratory function of myocardial tissue, myocardial cells and myocardial fibroblasts was determined. First, about 4 mg of cardiac tissue was cut and ground in PBS buffers, then centrifuged at 3500 rpm for 5 min. Tissues precipitated with mitochondrial respiratory solution (MiR05) were then suspended for the following mitochondrial respiratory function tests. The MiRO5 consisted of EGTA 0.5 mM, MgCl_2_·6 H_2_O 3 mM, Lactobionic acid 60 mM, Taurine 20 mM, KH_2_PO_4_ 10 mM, HEPES 20 mM, D‐sucrose 110 mM, BSA, and essentially fatty acid free 1 g l^−1^. Mitochondrial respiratory function was measured in a two‐chamber titration injection respirometer (Oxygraph‐2k; Oroboros Instruments, Innsbruck, Austria). Data were recorded through DatLab software 5.2 (Oroboros Instruments, Innsbruck, Austria). Reserve respiratory capacity was equal to the difference between maximum oxygen consumption rate and routine oxygen consumption rate. In permeabilized cells, the protocol with substrate–uncoupler–inhibitor titrations was used. After respiration was stabilized for a short time, routine respiration was measured. Glutamate (G, 5 mM) and malate (M, 2 mM) in the absence of ADP titration were used for inducing the respiratory leak state of complex I. Then, 5 mM ADP added was to detect the OXPHOS capacity of complex I. Maximal OXPHOS capacity was induced by succinate (Succ, 100 mM), including both complex I and complex II OXPHOS capacity. Next, oligomycin (Omy, 2.5 µM) and trifluoromethoxy carbonylcyanide phenylhydrazone (Fccp, 1 mM) titrations were used for the maximal uncoupled respiratory capacity of the electron transfer system. CII‐related uncoupled respiratory function was detected after the addition of rotenone (Rot, 0.5 µM). Finally, antimycin A (Ama, 2.5 µM) was given for residual oxygen consumption evaluation.

### Quantitative Real‐Time PCR

Total RNA was extracted using Trizol reagent (Invitrogen, USA) according to the manufacturer's instructions. Total RNA (0.5 µg) was reverse transcribed by TransScript reverse transcriptase (GMO) to obtain cDNA. RNA levels were measured using SYBR Green I incorporation on an ABI 7500 fast Real Time PCR system (Applied Biosystems, USA), and mRNA expression levels were calculated using the relative quantification 2^−△△CT^ method. The primers were synthesized by Invitrogen and listed in Table S2 (Supporting Information).

### Western Blot

Mouse heart tissue proteins were extracted for immunoblotting analysis. Total proteins were collected by treatment with RIPA lysis buffer (Beyotime, Beijing, China) and protease inhibitor mixture (Roche, Basel, Switzerland) at 4 °C, followed by centrifugation. Protein samples were separated by SDS‐PAGE and transferred to PVDF membranes. Membranes were blocked in Tris‐buffered saline containing 5% milk and then incubated with primary antibodies overnight at 4 °C. The primary antibodies include anti‐Cfp1 (1:1000, ab198977, Abcam, America), anti‐H3K4me3 (1:1000, 9751S, Cell Signaling Technology, America), anti‐H3K9ac (1:1000, 9649S, Cell Signaling Technology, America) and anti‐H3K27ac antibody (1:1000, 8173S, Cell Signaling Technology, America). The anti‐β‐actin (1:5000, 66009‐1‐Ig, Proteintech, America) and anti‐H3 (1:1000, 4499S, Cell Signaling Technology, America) were used as internal controls. Western blot bands were captured on an Odyssey Infrared imaging system (LI‐COR Biosciences, USA), and band intensities (area × OD) were measured in each group using Image StudioFull_5.2.5 software. Band intensities were standardized against internal controls. All antibodies were diluted in PBS buffer.

### Hematoxylin‐Eosin and Masson's Trichrome Staining

Mouse hearts were fixed in 4% paraformaldehyde, embedded in paraffin and sectioned. H&E and Masson's trichrome staining were performed according to standard procedures. Heart fibrosis was detected by Masson's trichrome staining. The size of fibrosis in each section was quantified using ImageJ software (National Institutes of Health) based on Masson trichrome staining.

### Chromatin Immunoprecipitation and Sequencing Analysis

Mouse hearts were used for ChIP‐seq analysis, with samples from at least three hearts per set. Cell Signaling SimpleChIP Plus Enzymatic Chromatin IP Kit (Magnetic Beads) #9005 kit was used. Cardiac tissue was cross‐linked with paraformaldehyde, and DNA was digested enzymatically to a length of approximately 150 to 900 bp. 1 µg of antibody was used for chromatin immunoprecipitation, and purified DNA was obtained by centrifugal column after enzymatic hydrolysis. Enriched DNA fragments were subjected to the preparation of standard libraries for ChIP‐seq according to the manufacturer's instructions, and the resulting ChIP‐seq libraries were sequenced on an Illumina Hi‐seq X‐Ten (Annoroad Gene Technology Co. Ltd). ChIP‐seq data were mapped to mouse genome (GRCm38) by using Bowtie2, and ChIP peaks were called using MACS2, with the input sample as the control. Signal track files in BigWig format were generated using the MACS2 pileup function and were normalized to 1 million reads for visualization. Deeptools was also used to plot the gene body and flanking region heatmap graph using the normalized signal intensity. MACS2 was used to call peaks, and followed by peak annotation using bedtools. Differential analysis between treat and control samples was conducted using bedtools. Functional analysis such as GO for differential peaks related genes was done by using in‐house scripts. The raw data from ChIP‐seq can be downloaded from the NCBI's SRA using accession number GSE241021.

### RNA Sequencing Analysis

The sampling instruments need to be sterilized by high temperature and autoclaving before use. After anesthesia, the mice were sacrificed by spinal cord transection, and the hearts were removed. The blood was drained in PBS buffer containing DEPC after precooling and then snap frozen in liquid nitrogen. Download reference from UCSC (ttp://hgdownload.soe.ucsc.edu/goldenPath/galGal4) gene and genome annotation file, use the Bowtie2 build reference genomic library, then through the TopHat clean data was mapped to a reference genome. At the same time, Bowtie2 was used for mapping and then compared with TopHat to make the mapping results more accurate. Differential gene expression analysis was performed using the DESeq software package. The significance of differential genes was calculated using DESeq software, and pairwise comparisons were made between all biological replicates of enriched/enhanced tissues (or group tissues) and all other tissues. Multiple test correction *p* value (FDR 5%) was used to determine whether genes were significantly differentially expressed. Raw data for RNA‐seq can be downloaded from SRA at NCBI using accession number GSE240852.

### Differentiation of Human Induced Pluripotent Stem Cells to Cardiomyocytes

Undifferentiated hiPSCs were purchased from NC5 (Help Stem Cell Innovations, NC2001) and cultured on Matrigel‐coated plates in an E8 medium (CA1001500, CELLAPY). Differentiation basal medium composed of RPMI1640 medium (C11875500BT, Thermo Fisher Scientific) and B27 minus insulin (A1895601, Thermo Fisher Scientific) was used to induce cardiomyocyte differentiation. Specifically, the 70∼80% confluent hiPSCs were incubated in differentiation basal medium added with CHIR‐99021 (HY‐10182, MCE) for 1 day and Wnt‐C59 (S7037, Selleck Chemicals) for 2 days. Then, the cells were cultured in RPMI1640 basal medium containing B27 (17 504 044, Thermo Fisher Scientific), which was replaced with fresh medium every 1∼2 days. Beating cells were observed after 8 days of differentiation induction and used for further study.

### Statistical Analysis

The data were expressed as mean ± SEM. Firstly, all data sets were tested for normality by D'Agostino & Pearson test (n ≥ 8) and Shapiro‐Wilk test (n < 8). Then, for normally distributed data, Student's t test (unpaired two‐tailed) was used to compare two groups; one‐way ANOVA followed by Dunnett multiple comparisons test was used to compare differences among multiple groups; For non‐normally distributed, the Mann‐Whitney U test (unpaired two‐tailed) was used for two groups. Survival curves were analyzed using Kaplan‐Meier univariate survival analysis. In the detection of mitochondrial respiratory function, Student's t test (paired two‐tailed) was used for the normal distribution, and Mann‐Whitney U test (paired two‐tailed) was used for the non‐normal distribution. All statistical analysis was performed with GraphPad Prism 9.4.1. A value of *P* < 0.05 was considered a statistically significant difference. (*, *P* < 0.05; **, *P* < 0.01; ***, *P* < 0.001; ****, *P* < 0.0001)

## Conflict of Interest

The authors declare no conflict of interests.

## Author Contributions

C.Z.L., Y.Z. and J.L.S. contributed equally to this study. C.Z.L., Y.J.L., Z.W.P. and B.Z.C. designed the research. C.Z.L., Y.Z. and J.L.S. supervised all aspects of the research. J.M.Y., K.Y.G, and Y.P. performed cellular experiments. H.R.B., Y.Z., D.S.L., Z.W.Z., and L.X.Z. conducted animal experiments. C.Z.L., S.J.L., Y.N.L., H.R.B. contributed to manuscript revision. C.Z.L., Y.J.L., Z.W.P. and B.Z.C. wrote and finalized the manuscript. All persons have made contributions to this work.

## Supporting information

Supporting Information

## Data Availability

The data that support the findings of this study are available in the supplementary material of this article.
