# Supplementary material for: Cfp1 Controls Cardiomyocyte Maturation by Modifying Histone H3K4me3 of Structural, Metabolic, and Contractile Related Genes
Source: Adv Sci (Weinh). 2024 Jan 9;11(11):2305992. doi: 10.1002/advs.202305992 (PMC10953565; doi:10.1002/advs.202305992)
Supplement: Supplementary file 1 — Supporting Information [file ADVS-11-2305992-s001.pdf]

ADVANCED SCIENCE
Open Access

Supporting Information

for *Adv. Sci.*, DOI 10.1002/advs.202305992

Cfp1 Controls Cardiomyocyte Maturation by Modifying Histone H3K4me3 of Structural, Metabolic, and Contractile Related Genes

Changzhu Li, Yang Zhang, Jingling Shen, Hairong Bao, Yue Zhao, Desheng Li, Sijia Li, Yining Liu, Jiming Yang, Zhiwen Zhou, Kangyi Gao, Lexin Zhao, Yao Pei, Yanjie Lu, Zhenwei Pan* and Benzhi Cai**

Supporting Information

Cfp1 Controls Cardiomyocyte Maturation by Modifying Histone H3K4me3 of Structural, Metabolic, and Contractile Related Genes

Changzhu Li [a], *Yang Zhang* [a], *Jingling Shen* [c], *Hairong Bao* [a], *Yue Zhao* [a], *Desheng Li* [a], *Sijia Li* [a], *Yining Liu* [a], *Jiming Yang* [a], *Zhiwen Zhou* [a], *Kangyi Gao* [a], *Lexin Zhao* [a], *Yao Pei* [a], *Yanjie Lu* [a, *], *Zhenwei Pan* [a, b, d, *], *Benzhi Cai* [a, *]

[a] Department of Pharmacology (State Key Laboratory of Frigid Zone Cardiovascular Disease, Key Laboratory of Cardiovascular Research, Ministry of Education), College of Pharmacy, Harbin Medical University, Harbin, Heilongjiang 150086, P. R. China;
[b] Research Unit of Noninfectious Chronic Diseases in Frigid Zone, Chinese Academy of Medical Sciences, 2019 Research Unit 070, Harbin, Heilongjiang 150086, P. R. China;
[c] Institute of Life Sciences, College of Life and Environmental Sciences, Wenzhou University, Wenzhou 325035, P.R. China;
[d] Key Laboratory of Cell Transplantation, The First Affiliated Hospital, Harbin Medical University.

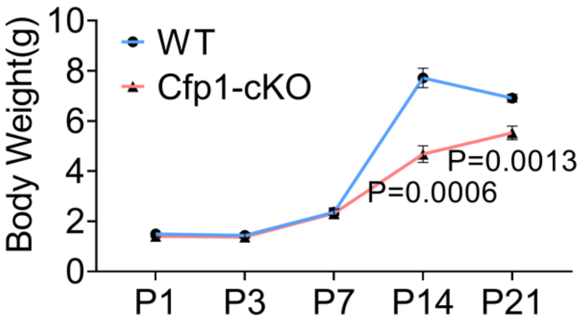

Figure S1. Body weight and cardiac function of Cfp1-cKO mice. Body weight of mice was recorded from postnatal days 1 to 21(P1-P21). n>5 per timepoint. *P* values are indicated on the graphs comparing WT vs Cfp1-cKO. Mann-Whitney U test was used. Bars represent the mean ± SEM.

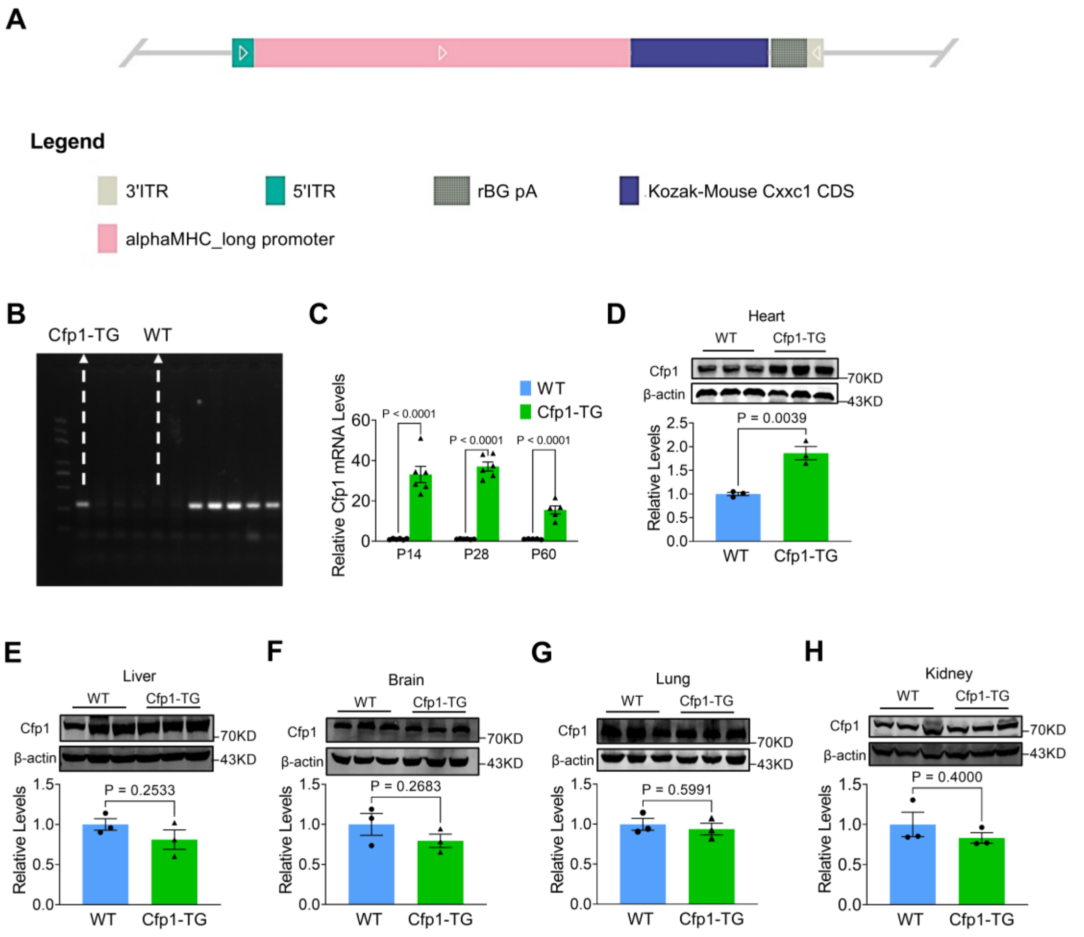

Figure S2. Construction of cardiac-specific Cfp1 transgenic overexpression mice. (A) Cfp1 protein-encoding cDNA was cloned downstream of cardiac-specific promoter α-MHC. (B) Qualitative analysis of transgenic mice by agarose gel electrophoresis. (C) qRT-PCR analyses of Cfp1 mRNA levels of Cfp1 in ventricles (P14, P28 and P60) from WT and Cfp1-TG mice. n=5-6. (D) Western blot analyses Cfp1 protein levels in ventricles from WT and Cfp1-TG mice. β-actin was used as loading control. n=3. (E-H) The expression level of Cfp1 protein in liver, brain, lung and kidney of transgenic mice by western blot. n=3. *P* values are indicated on the graphs comparing WT vs Cfp1-TG. Student's t test or Mann-Whitney U test was used. Bars represent the mean ± SEM.

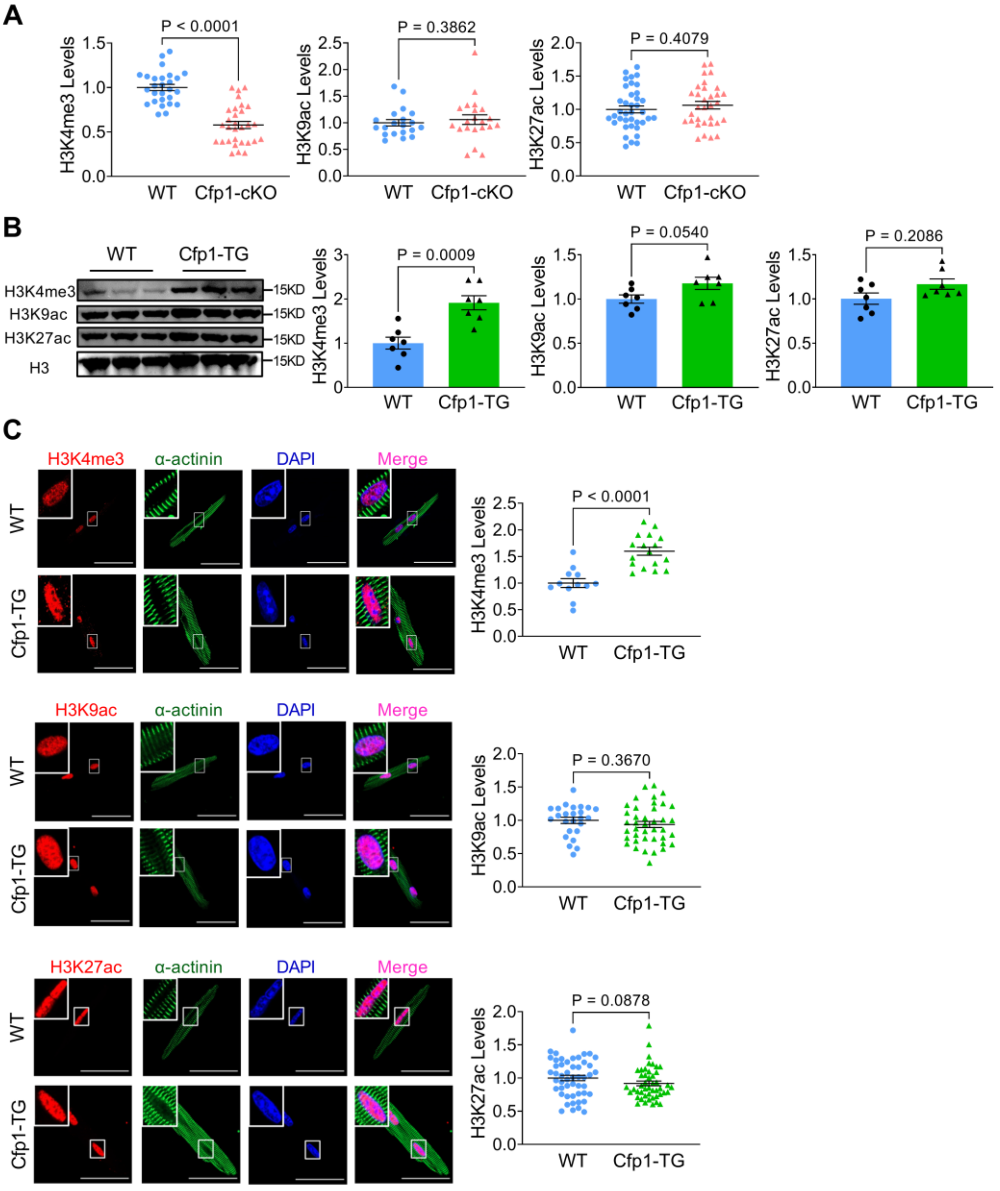

Figure S3. Levels of H3K4me3, H3K9ac, and H3K27ac in ventricles. (A) Quantification of H3K4me3, H3K9ac and H3K27ac levels in isolated cardiomyocytes from WT and Cfp1-cKO mice. n=20-38 cells from at least 3 mice. *P* values are indicated on the graphs comparing WT vs Cfp1-cKO. (B) Protein levels of H3K4me3, H3K9ac, H3K27ac in ventricles from WT and Cfp1-TG mice by western blot. H3 was used as loading control. n=7. Mice of 2 weeks old were used in the study. *P* values are indicated on the graphs comparing WT vs Cfp1-TG. (C) Immunofluorescent staining and quantification of H3K4me3, H3K9ac and H3K27ac levels in isolated cardiomyocytes from WT and Cfp1-TG mice. Scale bar: 50μm. n=12-50 cells from at least 3 mice. *P* values are indicated on the graphs comparing WT vs Cfp1-TG. Student's t test or Mann-Whitney U test was used. Bars represent the mean ± SEM.

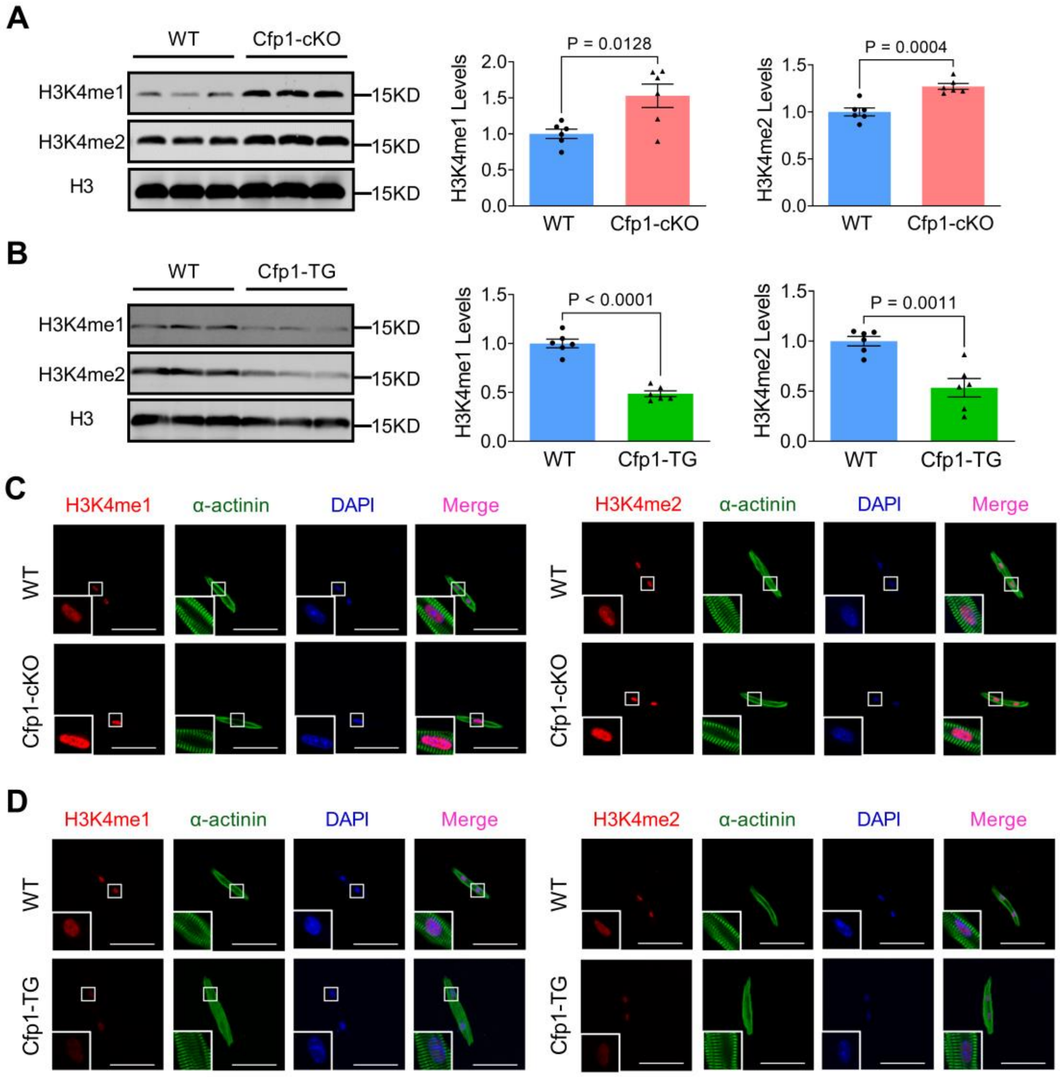

Figure S4. Levels of H3K4me1/2 in ventricles. (A) Protein levels of H3K4me1, H3K4me2 in ventricles from WT and Cfp1-cKO mice by western blot. H3 was used as loading control. n=6. *P* values are indicated on the graphs comparing WT vs Cfp1-cKO. Mice of 2 weeks old were used in the study. (B) Protein levels of H3K4me1, H3K4me2 in ventricles from WT and Cfp1-TG mice by western blot. H3 was used as loading control. n=6. *P* values are indicated on the graphs comparing WT vs Cfp1-TG. Mice of 2 weeks old were used in the study. (C) Immunofluorescent staining of H3K4me1/2 in isolated cardiomyocytes from WT and Cfp1-cKO mice. Scale bar: 50μm. n=7 cells from at least 3 mice. (D) Immunofluorescent staining of H3K4me1/2 in isolated cardiomyocytes from WT and Cfp1-TG mice. Scale bar: 50μm. n=7 cells from at least 3 mice. Student's t test was used. Bars represent the mean ± SEM.

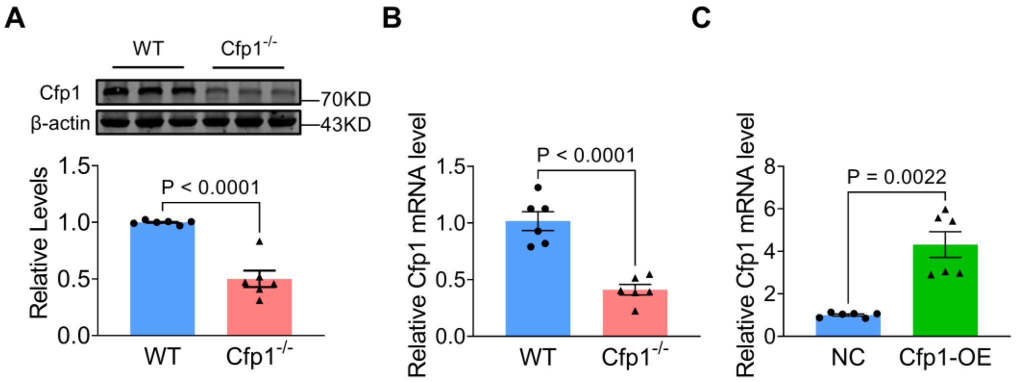

Figure S5. Protein and mRNA level of Cfp1. (A) Western blot analyses Cfp1 protein levels in ventricles from WT and Cfp1$^{-/-}$ mice. β-actin was used as loading control. n=6. *P* values are indicated on the graphs comparing WT vs Cfp1$^{-/-}$. (B) qRT-PCR analyses of Cfp1 mRNA levels of Cfp1 in ventricles from WT and Cfp1$^{-/-}$ mice hearts. n=6. *P* values are indicated on the graphs comparing WT vs Cfp1$^{-/-}$. (C) qRT-PCR was used to detect the expression level of Cfp1 mRNA in hiPSC-CMs. n=6. *P* values are indicated on the graphs comparing NC vs Cfp1-OE. Student's t test or Mann-Whitney U test was used. Bars represent the mean ± SEM.

Supplementary Tables

Table S1. Sequences of oligonucleotide primers used for PCR

Name	Primer Sequence
Cfp1$^{flox/flox}$-F	5'-CGAGAGATGAAGAGGAGCCA-3'
Cfp1$^{flox/flox}$-R	5'-CTGAATGGTCCTAGAACCTC-3'
Myh6-Cre or Myh6-MerCreMer -F	5'-GCCTGCATTACCGGTCGATGC-3'
Myh6-Cre or Myh6-MerCreMer -R	5'-CAGGGTGTTATAAGCAATCCC-3'
Cfp1-TG -F	5' - AGAGCCATAGGCTACGGTGTA-3'
Cfp1-TG -R	5'- TGATGTCCGGTTTGCGACAGA-3'

Table S2. Sequences of oligonucleotide primers used for qRT-PCR

Name	Primer Sequence
Human-Cfp1-F	5'-GCAAACCGGACATCAACTGC-3'
Human-Cfp1-R	5'-GCACTCCCGACAGTACCAC-3'
Human-Gja1-F	5'-GGTGACTGGAGCGCCTTAG-3'
Human-Gja1-R	5'-GCGCACATGAGAGATTGGGA-3'
Human-Myh6-F	5'-GCCCTTTGACATTCGCACTG-3'
Human-Myh6-R	5'-GGTTTCAGCAATGACCTTGCC-3'
Human-Myh7-F	5'-ACTGCCGAGACCGAGTATG-3'
Human-Myh7-R	5'-GCGATCCTTGAGGTTGTAGAGC-3'
Human-Myl2-F	5'-TTGGGCGAGTGAACGTGAAAA-3'
Human-Myl2-R	5'-CCGAACGTAATCAGCCTTCAG-3'
Human-Myl7-F	5'-GCCCAACGTGGTTCTTCCAA-3'
Human-Myl7-R	5'-CTCCTCCTCTGGGACACTC-3'
Human-Nppa-F	5'-CAACGCAGACCTGATGGATTT-3'
Human-Nppa-R	5'-AGCCCCCGCTTCTTCATTC-3'
Human-Nppb-F	5'-GAGGTCACTCCTATCCTCTGG-3'
Human-Nppb-R	5'-GCCATTTCCTCCGACTTTTCTC-3'
Human-Scn5a-F	5'-TCTCTATGGCAATCCACCCCA-3'
Human-scn5a-R	5'-GAGGACATACAAGGCGTTGGT-3'
Human-Tnni1-F	5'-CCGGAAGTCGAGAGAAAACCC-3'
Human-Tnni1-R	5'-TCAATGTCGTATCGCTCCTCA-3'

Human-Tnni3-F	5’-CTCTGCCAACTACCGAGCCTA-3’
Human-Tnni3-R	5’-CTCTTCTGCCTCTCGTTCCAT-3’
Human-Tpm1-F	5’-CTCCTCCTCTGGGACACTC-3’
Human-Tpm1-R	5’-GCCGACGTAGCTTCTCTGAAC-3’
Human-Tpm2-F	5’-TTTGGGCTCGACTCTCAATGA-3’
Human-Tpm2-R	5’-GGTGGCCGAGAGTAAATGTGG-3’
Mouse-Acsl1-F	5’-CGCACCCTTCCAACCAACA-3’
Mouse-Acsl1-R	5’-CGCTATTTCCACTGACTGCAT-3’
Mouse-Acsl3-F	5’-CTGCACAGGCGTGTTTTATGT-3’
Mouse-Acsl3-R	5’-ACGTGGGACCAAAGAGACTAT-3’
Mouse-Atp2a2-F	5’-GAGAACGCTCACACAAAGACC-3’
Mouse-Atp2a2-R	5’-ACTGCTCAATCACAAGTTCCAG-3’
Mouse-Atp5b-F	5’-CCCTTCTGCTGTGGGCTATC-3’
Mouse-Atp5b-R	5’-AAACGTAGTAGCAGCAGGGGCAG-3’
Mouse-Atp5g3-F	5’-TGCATCAGTATTATCTCGACCAG-3’
Mouse-Atp5g3-R	5’-GCACCTGCACCAATGAATTT-3’
Mouse-Ckmt2-F	5’-AGACAAGCATAAGACCGACCT-3’
Mouse-Ckmt2-R	5’-AGGCAGAGTGTAACCCTTGAT-3’
Mouse-Nusap1-F	5’-AGTTCCTTCTCTGCCGGAGC-3’
Mouse-Nusap1-R	5’-GCTTCTTAAAGTTTGGTGTTGTGGC-3’
Mouse-Kif22-F	5’-GTGTTGCTGTCCCACTAGGT-3’
Mouse-Kif22-R	5’-AAGAGGAACGTGCGTTAGCA-3’
Mouse-Aurka-F	5’-CACTTCCGGGCTCCGACA-3’
Mouse-Aurka-R	5’- ACATCTGTCCATGTCACCGTAAA-3’
Mouse-Cox6a2-F	5’-CCAGAGTTCATCCCGTATCACC-3’
Mouse-Cox6a2-R	5’-GATTGTGGAAAAGCGTGTGGT-3’
Mouse-Cpt1b-F	5’-GACTTCCGGCTTAGTCGGG-3’
Mouse-Cpt1b-R	5’-GAATAAGGCGTTTCTTCCAGGA-3’
Mouse-Eno2-F	5’-AAGGGATGGGGACAAACAGC-3’
Mouse-Eno2-R	5’-CAATGTGGCGATAGAGGGGC-3’
Mouse-Kcnj2-F	5’-CAACCGCTACAGCATCGT-3’
Mouse-Kcnj2-R	5’-GCACTGTTGTCGGGTATG-3’
Mouse-Gata4-F	5’-CACCCCAATCTCGATATGTTTGA-3’

Mouse-Gata4-R	5’-GCACAGGTAGTGTCCCGTC-3’
Mouse-Gja1-F	5’-CTGAGTGCGGTCTACACCTG-3’
Mouse-Gja1-R	5’-GAGCGAGAGACACCAAGGAC-3’
Mouse-Hk2-F	5’-CACGGAGCTCAACCAAAACC-3’
Mouse-Hk2-R	5’-TTACTCGGAGCACACGGAAG-3’
Mouse-Mef2c-F	5’-ATCCCGATGCAGACGATTCAG-3’
Mouse-Mef2c-R	5’-AACAGCACACAATCTTTGCCT-3’
Mouse-Mfn1-F	5’-ATGGCAGAAACGGTATCTCCA-3’
Mouse-Mfn1-R	5’-GCCCTCAGTAACAAACTCCAGT-3’
Mouse-Mki67-F	5’-ATCATTGACCGCTCCTTTAGGT-3’
Mouse-Mki67-R	5’-GCTCGCCTTGATGGTTCCT-3’
Mouse-Myh6-F	5’-TGAGTGGGAGTTTATCGACTTCG-3’
Mouse-Myh6-R	5’-CCTTGACATTGCGAGGCTTC-3’
Mouse-Myh7-F	5’-AGACTGTCAACACTAAGAGGGT-3’
Mouse-Myh7-R	5’-TGCCCCAAAATGGATTCGGAT-3’
Mouse-Myl2-F	5’-CATTCTCAACGCATTCAAGGTG-3’
Mouse-Myl2-R	5’-TGCGAACATCTGGTCGATCTC-3’
Mouse-Myl4-F	5’-AGAAACCCGAGCCTAAGAAGG-3’
Mouse-Myl4-R	5’-TGGAGTCCGGTCAAACAATGA-3’
Mouse-Myl7-F	5’-AGAAACCCGAGCCTAAGAAGG-3’
Mouse-Myl7-R	5’-TGGAGTCCGGTCAAACAATGA-3’
Mouse-Mylcd-F	5’-GCACGTCCGGGAAATGAAC-3’
Mouse-Mylcd-R	5’-GCCTCACACTCGCTGATCTT-3’
Mouse-Ndufb5-F	5’-CCTGGATACCGCAGCTAGGA-3’
Mouse-Ndufb5-R	5’-GCGGCGCAATACGAATGCCC-3’
Mouse-Nppb-F	5’-GAGGTCACTCCTATCCTCTGG-3’
Mouse-Nppb-R	5’-GCCATTTCCTCCGACTTTTCTC-3’
Mouse-Ppargc1a-F	5’-TATGGAGTGACATAGAGTGTGCT-3’
Mouse-Ppargc1a-R	5’-GTCGCTACACCACTTCAATCC-3’
Mouse-Ryr2-F	5’-GCCACCGGACACTCCTCTAT-3’
Mouse-Ryr2-R	5’-CCAACACGCACTTTTTCTCCT-3’
Mouse-Scn5a-F	5’-TGCTGAATAAGGGCAAAACCA-3’
Mouse-scn5a-R	5’-GCTGAAGAGCGAATGTACCAAAA-3’

Mouse-Tnni1-F	5’-ATGCCGGAAGTTGAGAGGAAA-3’
Mouse-Tnni1-R	5’-TCCGAGAGGTAACGCACCTT-3’
Mouse-Tnni3-F	5’-CTCTGCCAACTACCGAGCCTA-3’
Mouse-Tnni3-R	5’-CTCTTCTGCCTCTCGTTCCAT-3’
Mouse-Tpm1-F	5’-AACGGTGACGAACAACTTGAA-3’
Mouse-Tpm1-R	5’-GGAAGTCATATCGTTGAGAGCG-3’
Mouse-Tpm2-F	5’-CGGGCCATGAAGGATGAGG-3’
Mouse-Tpm2-R	5’-CCCACATTTACTCTCAGCCAC-3’
18s-F	5’-CCTGGATACCGCAGCTAGGA-3’
18s-R	5’-GCGGCGCAATACGAATGCCC-3’

RNA-seq: To review GEO accession GSE240852: Go to https://www.ncbi.nlm.nih.gov/geo/query/acc.cgi?acc=GSE240852; enter token slqtkuckdpihxyb into the box.

ChIP-seq: To review GEO accession GSE241021: Go to https://www.ncbi.nlm.nih.gov/geo/query/acc.cgi?acc=GSE241021; enter token kzcdqkkcbbqrpqd into the box.
